# Corrigendum to “Do children with disabilities have the same opportunities to play as children without disabilities? Evidence from the multiple indicator cluster surveys in 38 low and middle-income countries” [ECLINM 67 (2023) 102361]

**DOI:** 10.1016/j.eclinm.2024.102686

**Published:** 2024-06-19

**Authors:** Tracey Smythe, Shanquan Chen, Sara Rotenberg, Marianne Unger, Emily Miner, Frederic Seghers, Chiara Servili, Hannah Kuper

**Affiliations:** a International Centre for Evidence in Disability, London School of Hygiene & Tropical Medicine, Keppel St, London WC1E 7HT, UK; b Division of Physiotherapy, Department of Health and Rehabilitation Sciences, Stellenbosch University, Cape Town 8000, South Africa; c Nuffield Department of Primary Care Health Sciences, Medical Sciences Division, University of Oxford, OX2 6GG, UK; d Clinton Health Access Initiative, Boston, MA 02127, USA; e Department of Mental Health and Substance Abuse, World Health Organization, Geneva, Switzerland

In the article titled “Do children with disabilities have the same opportunities to play as children without disabilities? Evidence from the multiple indicator cluster surveys in 38 low and middle-income countries” published in Lancet eClinicalMedicine, Vol. 67, No. 102361, the authors identified an error in the reported percentage reduction in play opportunities for children with disabilities.

## Description of the error

In the abstract, it was noted that adjusted analyses show that on average, children with disabilities had approximately 9% fewer play opportunities than those without disabilities (adjusted RR [aRR] = 0.88, 95% CI = 0.82–0.93). The correct percentage reduction should be 12%.

## Explanation of how the error occurred

The error occurred during the final stages of manuscript preparation. While the aRR was updated to 0.88 after peer review comments were addressed, the text describing the percentage reduction in play opportunities was inadvertently not updated to reflect this change. The correct interpretation of an aRR of 0.88 is that children with disabilities have approximately 12% fewer play opportunities than those without disabilities.

## Impact on main results

The correction of this percentage does not alter the main results or conclusions of the paper. The key finding remains that children with disabilities have significantly fewer play opportunities compared to those without disabilities. The corrected statement should read:

Children with disabilities had approximately 12% fewer play opportunities than those without disabilities (adjusted RR [aRR] = 0.88, 95% CI = 0.82–0.93).”

The authors apologise for any confusion this error may have caused and appreciate the understanding of the readers.

