# Supplementary material for: Do children with disabilities have the same opportunities to play as children without disabilities? Evidence from the multiple indicator cluster surveys in 38 low and middle-income countries
Source: eClinicalMedicine. 2023 Dec 12;67:102361. doi: 10.1016/j.eclinm.2023.102361 (PMC10758749; doi:10.1016/j.eclinm.2023.102361)
Supplement: Supplementary Material [file mmc1.docx]

**Supplementary Material**.

Appendix

Figure 1: flowchart of selection of participants for 212,194 children aged 2-4years from 38 Multiple Indicator Cluster Survey (MICS) countries, 2017 - 202


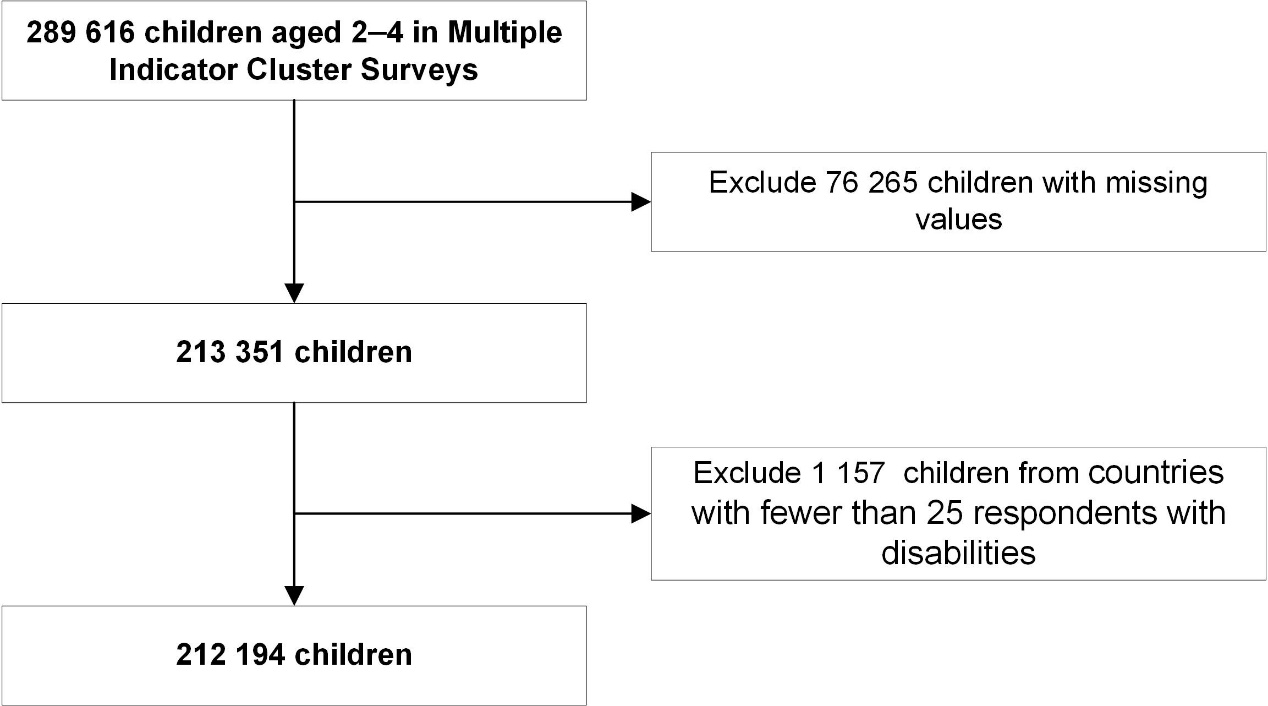


Appendix 2: Opportunities for play definition

Engagement of all four activities in the last three days: Activities include telling stories; singing songs to or with the child; taking the child outside the home; and playing with the child

Example question:

| **EC5**. In the past 3 days, did you or any household member age 15 or over engage in any of the following activities with (***name***):  *If ‘Yes’, ask:*  Who engaged in this activity with (***name***)?  *A foster/step mother or father living in the household who engaged with the child should be coded as mother or father.*  *Record all that apply.*  *‘No one’ cannot be recorded if any household member age 15 and above engaged in activity with child.* |
| --- |
|  |
|  |
| [B] Told stories to (***name***)? |
| [C] Sang songs to or with (***name***),  including lullabies? |
| [D] Took (***name***) outside the home? |
| [E] Played with (***name***)? |
|  |

Appendix 3: Adjusted risk ratios of play opportunities for boys and girls with disabilities aged 2-4years from 38 Multiple Indicator Cluster Survey (MICS) countries, 2017 - 202

| **Variable** | **Play Opportunities** | **Play opportunities with mother** | **Play opportunities with father** | **Play opportunities with other people** |
| --- | --- | --- | --- | --- |
| Boys with disability | Reference | Reference | Reference | Reference |
| Girls with disability | 0.97 [0.90, 1.03] | 0.90 [0.78, 1.03] | **7.80 [1.14, 53.36] ^&^** | 1.92 [0.83, 4.44] |

^&^ p < 0.05

Appendix 4: Adjusted risk ratios for opportunities for play with other people disaggregated by country according to disability status in children aged 2-4years from 38 Multiple Indicator Cluster Survey (MICS) countries, 2017 - 2020


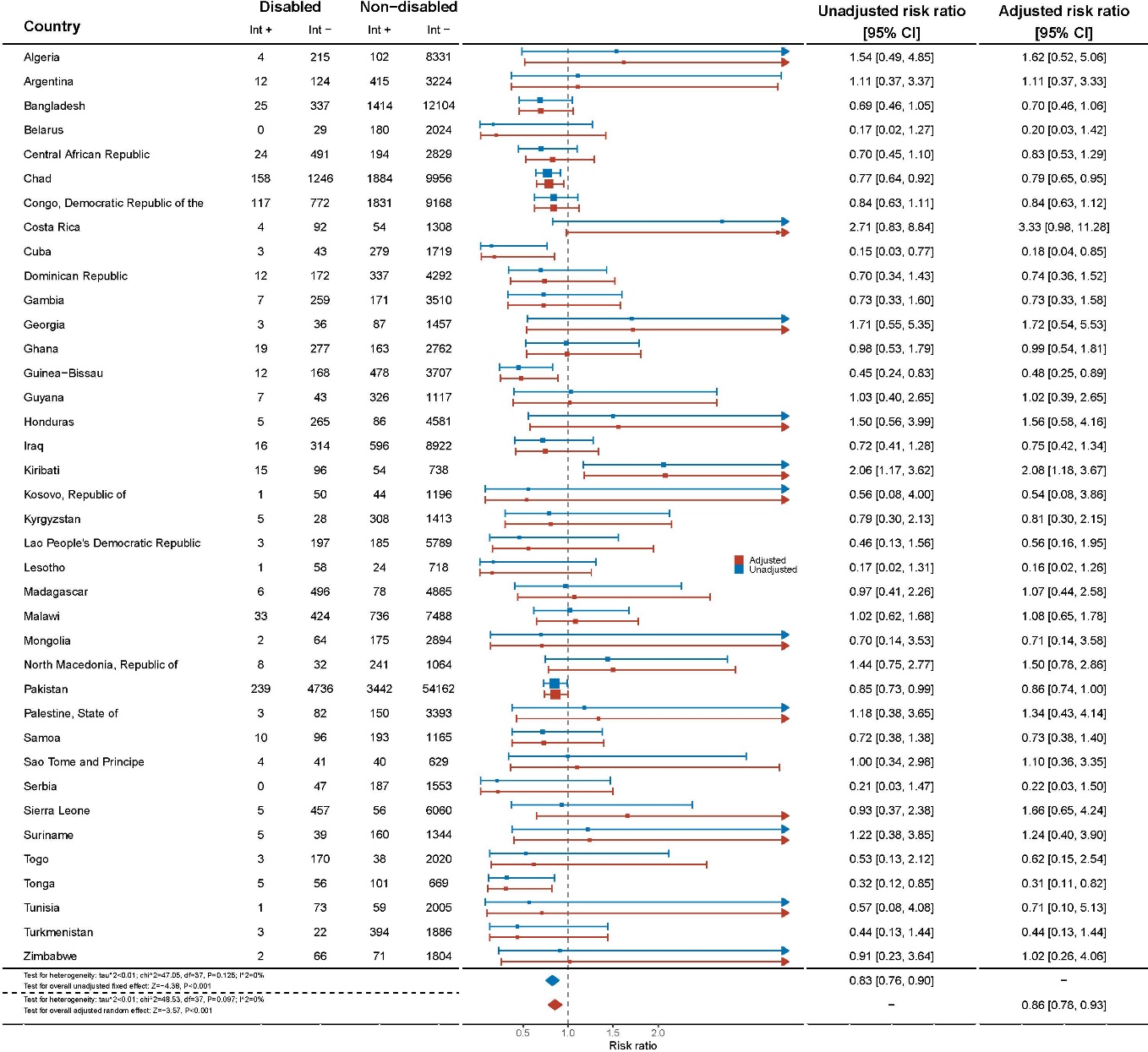


**Appendix 5: Sensitivity analysis**

Sensitivity analysis Table 5.1: Adjusted risk ratio for play opportunities experienced by children (2-4 years) with disabilities from 38 Multiple Indicator Cluster Survey (MICS) countries, 2017 - 2020

| **Outcomes** | **Overall adjusted RR** | **Girl adjusted RR** | **Boy adjusted RR** |
| --- | --- | --- | --- |
| **Number of books** |  |  |  |
| **books >= 3** | **0.95 [0.90, 1.00] &** | 0.97 [0.88, 1.08] | 0.56 [0.28, 1.14] |
| **books >= 10** | 0.67 [0.33, 1.34] | 2.13 [0.69, 6.57] | 0.71 [0.32, 1.53] |
| **Has toy** | **0.97 [0.95, 0.99] &** | **0.97 [0.94, 1.00] &** | 0.98 [0.95, 1.00] |
| **Play overall (at least 4 times)** | **0.89 [0.85, 0.94] &** | **0.89 [0.84, 0.95] &** | **0.90 [0.84, 0.97] &** |
| Sing song | 0.98 [0.96, 1.01] | 1.00 [0.97, 1.02] | 0.99 [0.96, 1.03] |
| Tell story | **0.90 [0.86, 0.94] &** | **0.89 [0.84, 0.95] &** | **0.92 [0.87, 0.97] &** |
| Take outside | 0.99 [0.96, 1.01] | 1.00 [0.98, 1.03] | 0.98 [0.95, 1.01] |
| Play with the child | 1.00 [0.98, 1.01] | 0.99 [0.96, 1.01] | **1.02 [1.00, 1.03] &** |
| **Play opportunities with mother** | **0.87 [0.79, 0.96] &** | **0.83 [0.72, 0.96] &** | 0.58 [0.29, 1.18] |
| **Play opportunities with father** | 0.85 [0.71, 1.02] | 0.55 [0.23, 1.32] | 0.51 [0.23, 1.11] |
| **Play opportunities with other people** | **0.83 [0.77, 0.89] &** | **0.82 [0.74, 0.91] &** | **0.83 [0.75, 0.91] &** |

^&^ p < 0.05

Sensitivity analysis Table 5.2: Adjusted risk ratios forplay experiences (disaggregated by impairment type with children without disability as reference) for 212,194 children aged 2-4years from 38 Multiple Indicator Cluster Survey (MICS) countries, 2017 - 2020

| **impairment** | **Opportunity to play overall [95%CI]** | **Mother provides play [95%CI]** | **Father provides play [95%CI]** | **Other people provide play [95%CI]** |
| --- | --- | --- | --- | --- |
| **Seeing** | 1.14 [0.98, 1.32] | 0.82 [0.45, 1.48] | **2.32 [1.55, 3.51] &** | 0.48 [0.15, 1.52] |
| **Hearing** | 0.62 [0.36, 1.08] | 0.63 [0.28, 1.45] | **3.92 [2.76, 5.62] &** | 1.59 [0.77, 3.29] |
| **Walking** | **0.53 [0.29, 0.99] &** | 0.65 [0.36, 1.17] | 1.17 [0.60, 2.29] | 0.91 [0.47, 1.76] |
| **Fine motor** | **0.41 [0.19, 0.91] &** | 0.56 [0.25, 1.26] | 1.54 [0.61, 3.87] | 0.95 [0.39, 2.31] |
| **Communication** | **0.70 [0.62, 0.78] &** | **0.68 [0.59, 0.80] &** | 0.39 [0.15, 1.00] | **0.37 [0.15, 0.93] &** |
| **Learning** | **0.73 [0.66, 0.81] &** | **0.66 [0.56, 0.79] &** | 0.54 [0.25, 1.15] | **0.49 [0.24, 0.99] &** |
| **Play** | **0.83 [0.73, 0.95] &** | 1.00 [0.85, 1.17] | **1.85 [1.34, 2.55] &** | **1.34 [1.12, 1.81] &** |
| **Behaviour** | 1.04 [0.97, 1.11] | 0.99 [0.88, 1.12] | 1.17 [0.93, 1.47] | 1.07 [0.91, 1.25] |

^&^ p < 0.05

Sensitivity analysis Figure 5.1: Adjusted risk ratios by country for play opportunities according to disability status in children aged 2-4years from 38 Multiple Indicator Cluster Survey (MICS) countries, 2017 - 2020 *.


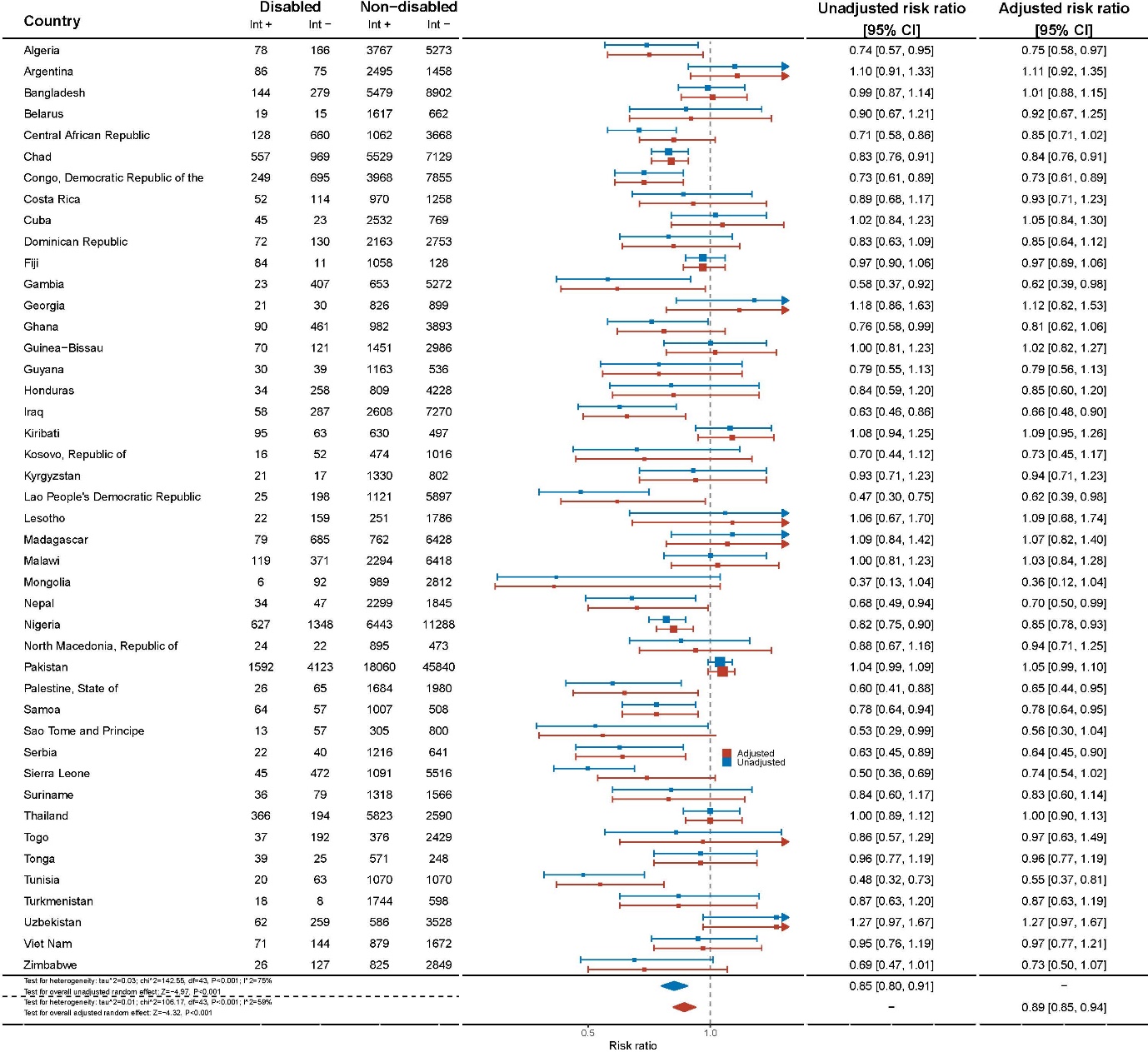


*Data were extracted from weighted logistic regression models with ‘opportunity to play’ as the outcome and disability status as the key predictor, controlling for age, sex, and wealth status.

Sensitivity analysis Figure 5.2: Adjusted risk ratios for opportunities to play provided by mother disaggregated by country according to disability status in children aged 2-4years from 38 Multiple Indicator Cluster Survey (MICS) countries, 2017 - 2020
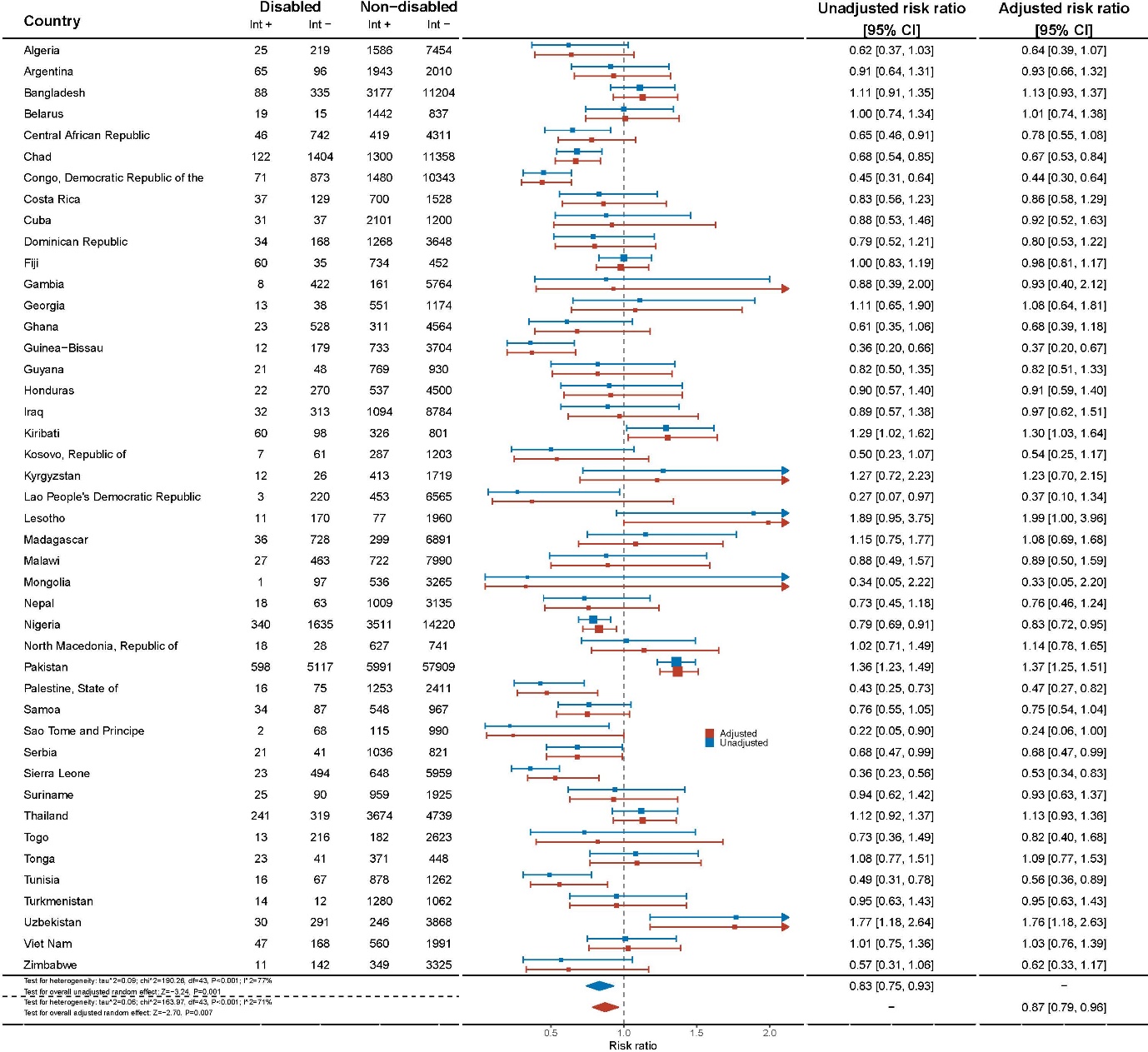


Sensitivity analysis Figure 5.3: Adjusted risk ratios for opportunities to play provided by father disaggregated by country according to disability status in children aged 2-4years from 38 Multiple Indicator Cluster Survey (MICS) countries, 2017 - 2020
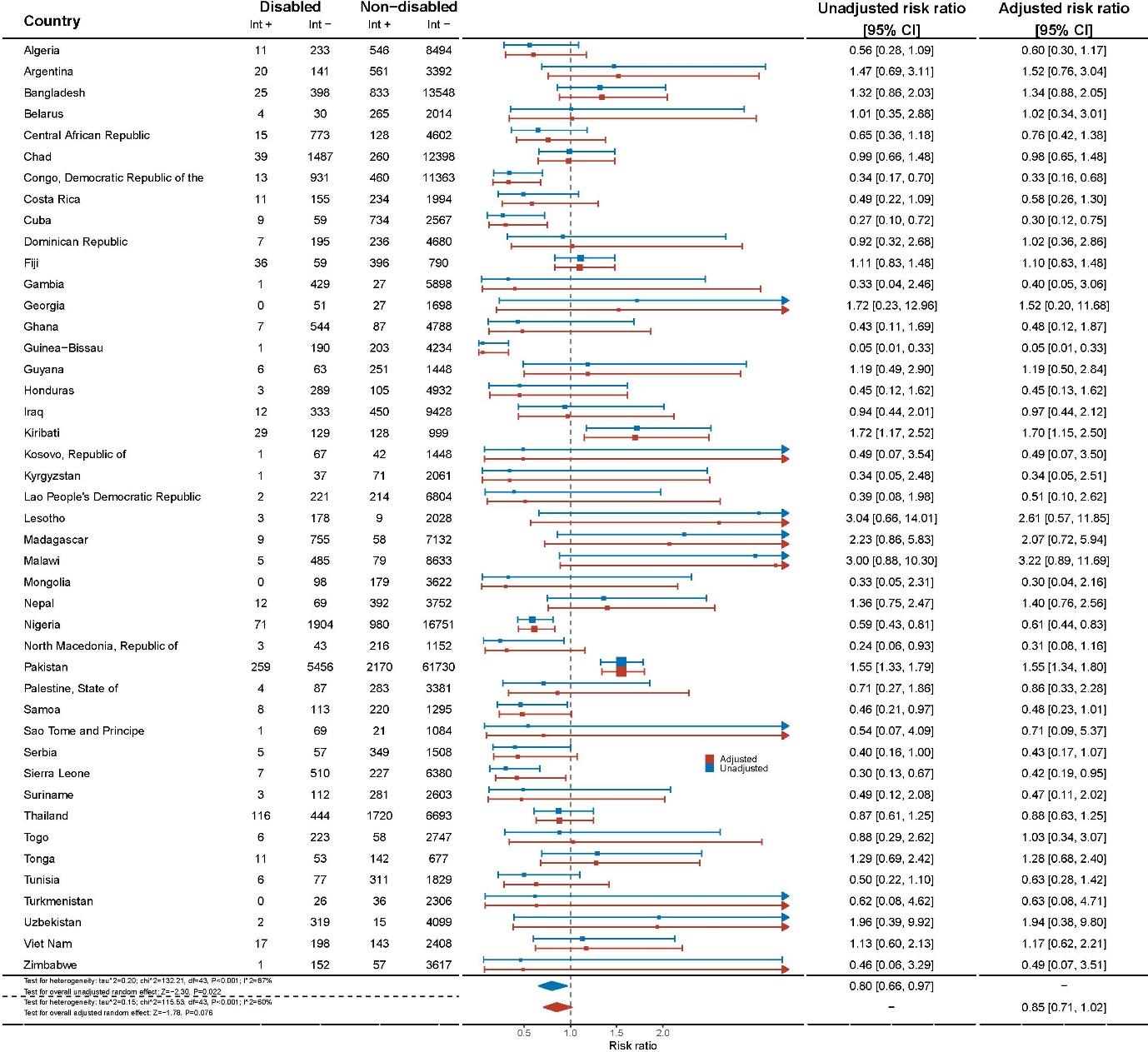


Sensitivity analysis Fig 5.4: Adjusted risk ratios for opportunities for play with other people disaggregated by country according to disability status in children aged 2-4years from 38 Multiple Indicator Cluster Survey (MICS) countries, 2017 - 2020

**
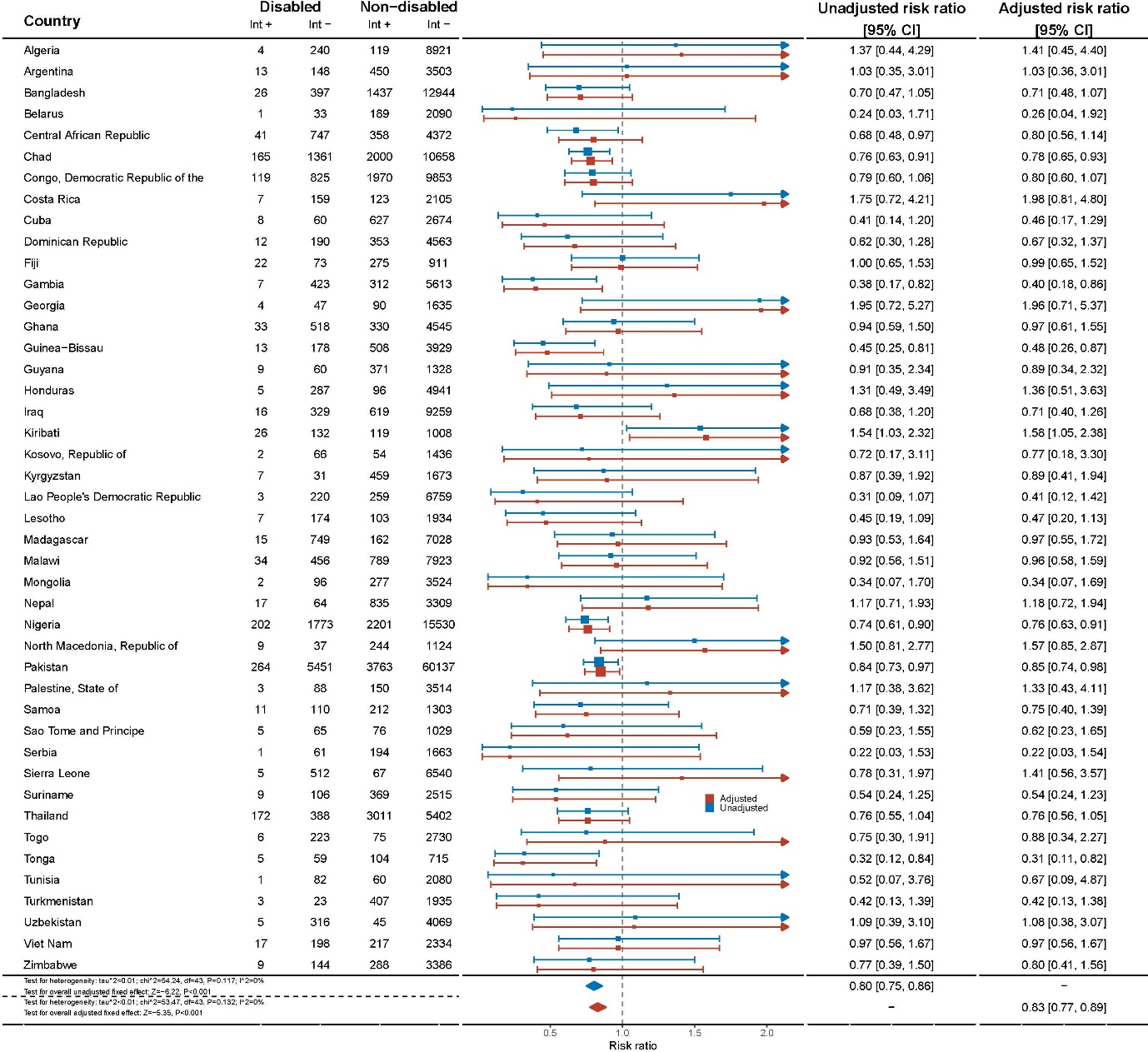
**
